# Role of D(−)-Lactic Acid in Prevention of *Chlamydia trachomatis* Infection in an In Vitro Model of HeLa Cells

**DOI:** 10.3390/pathogens12070883

**Published:** 2023-06-28

**Authors:** Chiara Zalambani, Nicola Rizzardi, Giacomo Marziali, Claudio Foschi, Sara Morselli, Marielle Ezekielle Djusse, Marina Naldi, Romana Fato, Natalia Calonghi, Antonella Marangoni

**Affiliations:** 1 Department of Pharmacy and Biotechnology (FABIT), University of Bologna, 40126 Bologna, Italy; chiara.zalambani2@unibo.it (C.Z.); nicola.rizzardi2@unibo.it (N.R.); giacomo.marziali3@unibo.it (G.M.); marina.naldi@unibo.it (M.N.); romana.fato@unibo.it (R.F.); natalia.calonghi@unibo.it (N.C.); 2 Microbiology, Department of Medical and Surgical Sciences (DIMEC), University of Bologna, 40138 Bologna, Italy; sara.morselli6@unibo.it (S.M.); djussemarielle@yahoo.fr (M.E.D.); antonella.marangoni@unibo.it (A.M.); 3 Microbiology Unit, IRCCS Azienda Ospedaliero-Universitaria di Bologna, 40138 Bologna, Italy

**Keywords:** *Chlamydia trachomatis*, lactic acid isomers, *Lactobacillus crispatus*, *Lactobacillus reuteri*, histone acetylation and lactylation, gene expression, oxygen consumption rate (OCR)

## Abstract

A vaginal microbiota dominated by certain *Lactobacillus* species may have a protective effect against *Chlamydia trachomatis* infection. One of the key antimicrobial compounds produced is lactic acid, which is believed to play a central role in host defense. *Lactobacillus* strains producing the D(−)-lactic acid isomer are known to exert stronger protection. However, the molecular mechanisms underlying this antimicrobial action are not well understood. The aim of this study was to investigate the role of D(−)-lactic acid isomer in the prevention of *C. trachomatis* infection in an in vitro HeLa cell model. We selected two strains of lactobacilli belonging to different species: a vaginal isolate of *Lactobacillus crispatus* that releases both D(−) and L(+) isomers and a strain of *Lactobacillus reuteri* that produces only the L(+) isomer. Initially, we demonstrated that *L. crispatus* was significantly more effective than *L. reuteri* in reducing *C. trachomatis* infectivity. A different pattern of histone acetylation and lactylation was observed when HeLa cells were pretreated for 24 h with supernatants of *Lactobacillus crispatus* or *L. reuteri*, resulting in different transcription of genes such as CCND1, CDKN1A, ITAG5 and HER-1. Similarly, distinct transcription patterns were found in HeLa cells treated with 10 mM D(−)- or L(+)-lactic acid isomers. Our findings suggest that D(−) lactic acid significantly affects two non-exclusive mechanisms involved in *C. trachomatis* infection: regulation of the cell cycle and expression of EGFR and α5β1-integrin.

## 1. Introduction

*Chlamydia trachomatis* (CT), an obligate intracellular pathogen, is the causative agent of the most common bacterial sexually transmitted infection (STI) worldwide with significant clinical and economic impact [1]. During its developmental cycle, CT alternates between two functionally and morphologically distinct forms: the extracellular form known as the infectious elementary body (EB) and the intracellular, noninfectious and replicating form known as the reticular body (RB). Since a large proportion of urogenital chlamydial infections in women are asymptomatic, they are at high risk of not being treated, leading to various complications and sequelae such as pelvic inflammatory disease, infertility and ectopic pregnancy [2].

A vaginal microbiota dominated by lactobacilli is crucial for the prevention of CT infections [3,4,5], although there are large differences depending on the species [6,7,8]. The protective role of lactobacilli against CT is exerted by several mechanisms acting on both the extracellular and intracellular steps of the cycle [9,10,11], including changes in lipid composition and exposure of the α5-integrin subunit in the plasma membrane of epithelial cells [12].

Lactobacilli provide protection against pathogens in several ways, including lactic acid (LA), short-range bacteriocins and long-range hydrogen peroxide (H_2_O_2_) production. It is believed that LA plays a central role in host defense, and it is important to emphasize that there are two isomeric forms of LA, D(−) and L(+) (D(−)-LA and L(+)-LA) [9,13,14]. The presence of all these compounds, whether independently or in combination, influences the host’s susceptibility to CT infection, highlighting the significance of the host–microbiota relationship.

Since both D(−) and L(+) isomers are present in vaginal fluid and their relative amounts depend on which lactobacillus species predominates [15,16,17], we decided to conduct a comprehensive study to investigate the role of the two LA isomers in the reduction in CT infectivity.

To this end, using an in vitro HeLa cell model, we investigated the protective role of two different isolates of vaginal lactobacilli, namely *Lactobacillus crispatus* (*L. crispatus*), which releases both D(−) and L(+) isomers, and *Lactobacillus reuteri* (*L. reuteri*), which produces only the L(+) isomer. Our goal was to investigate the effects of LA in the context of several non-exclusive mechanisms, such as host energy resources, epigenetic changes and cell-cycle regulation. The depletion of energy resources in host cells is critical for both EB infection and subsequent transformation into RBs. In addition, epigenetic changes can trigger expression of various genes involved in both cell-cycle regulation and surface plasma membrane protein modification. To investigate these points, we analyzed the effect of cell-free supernatants of lactobacilli supplemented with different amounts of D(−)-LA or L(+)-LA on the viability of HeLa cells. Subsequently, cellular metabolic status was assessed by oxygen consumption rate (OCR). Finally, we analyzed both the acetylation and lactylation status of histones from HeLa cells treated with *L. crispatus* or *L. reuteri* supernatants.

## 2. Materials and Methods

### 2.1. Lactobacilli Strains and Growth Conditions

Lactobacilli were isolated during routine diagnostic procedures from vaginal samples submitted to the Microbiology Unit of Sant’Orsola-Malpighi Hospital of Bologna (Italy), using de Man, Rogosa and Sharpe (MRS) agar plates (Difco, Detroit, MI, USA) in anaerobic conditions. For bacterial identification, MALDI-TOF MS analysis (Bruker Daltonics, Bremen, Germany) was performed by using direct analysis of bacterial colonies as previously detailed [18]. For further experiments, two different isolates were used, one belonging to *L. crispatus* species and the other to *L. reuteri* species, identified by MALDI-TOF MS with scores > 2.0, and glycerol stock preparations were stored at −80 °C until use. *L. crispatus* and *L. reuteri* used throughout all experiments were grown in MRS medium supplemented with 0.05% L-cysteine, at 37 °C for 24 h in anaerobic jars supplemented with GasPak EZ (Sparks, MD, USA). The turbidity of 24 h lactobacilli cultures was adjusted to an optical density (OD_600nm_) of 2, corresponding to a cell concentration of 5 × 10^8^ colony-forming units (CFU)/mL. Cell suspensions were centrifuged at 5000× *g* for 10 min at 4 °C, then supernatants were filtered through a 0.2 μm membrane filter to obtain cell-free supernatants.

### 2.2. Cell Culture

HeLa cells, a human cervical adenocarcinoma epithelial cell line (ATCC^®^ CCL-2), were seeded at 2 × 10^4^ cells/cm^2^ in a petri plastic plate (Orange Scientific, Braine-l’Alleud, Belgium) and cultured in 5% CO_2_ at 37 °C in Dulbecco’s modified Eagle medium (D-MEM) (Labtek Eurobio, Milan, Italy), supplemented with 10% FBS (Euroclone, Milan, Italy) and 2 mM L-glutamine (Sigma-Aldrich, Milan, Italy).

### 2.3. CT Propagation and Titration

CT GO/86 (serovar D), isolated during routine diagnostic procedures in the Microbiology Unit of Sant’Orsola-Malpighi Hospital of Bologna (Italy) and belonging to the laboratory collection, was used throughout all experiments. CT was propagated in HeLa cells, cultured in Dulbecco’s minimal essential medium (DMEM), and supplemented with 10% fetal bovine serum, 1% L-glutamine 200 mM and antibiotics (vancomycin 10 mg/L, gentamicin 10 mg/L and amphotericin B 0.3 mg/L). Purification of EBs and evaluation of the infectivity titer (expressed as inclusion-forming units, IFU/mL) have been described in detail, elsewhere [19].

### 2.4. Effect of Lactobacilli on CT Infectivity

HeLa cells were seeded at 2 × 10^4^ cells/cm^2^ in plastic wells (Orange Scientific, Braine-l’Alleud, Belgium) or on sterile glass coverslips for 48 h and treated with *L. crispatus* or *L. reuteri* at a ratio of 1:100 or 1:200 (HeLa cells: *Lactobacillus*) for 1 h at 37 °C and 5% CO_2_ atmosphere. Afterwards, HeLa bacteria co-cultures were washed 3 times with PBS to remove unbound bacteria and then were treated with 5 × 10^4^ CT EBs, corresponding to multiplicity of infection (MOI) 1 for 1 h at 37 °C and 5% CO_2_ atmosphere. Samples were washed 3 times with PBS, fixed in 3% paraformaldehyde for 10 min and marked with a monoclonal antibody against the chlamydial membrane lipopolysaccharide antigen conjugated with fluorescein (Meridian, Cincinnati, OH, USA) for 30 min at RT. The bacterial cells were centrifuged at 10,000× *g*, and the 1:100 diluted supernatant was sterile-filtered through a 0.2 µm filter and stored at −20 °C until use. The D(−)-LA or L(+)-LA enriched *L. reuteri* supernatant was prepared by adding 10 mM of D(−)-LA or L(+)-LA. HeLa cells were incubated with the enriched supernatant solutions for 1 h at 37 °C with 5% CO_2_. After incubation, the different samples cells were infected with CT EB for 48 h.

The number of IFUs was counted in 60 randomly chosen 100× microscopic fields. The results were expressed as a percentage (median percentage ± median absolute deviation) of CT infectivity, comparing the number of IFUs in the individual samples with the control slides (set at 100%).

### 2.5. Lactate Dehydrogenase Activity

Bacterial lysates for lactate dehydrogenase activity measurement were prepared diluting 5 × 10^8^ CFU/mL of the sub-cultured bacterial culture of lactobacilli with PBS to OD_600nm_ = 0.1, centrifuged at 4 °C and 12,000× *g* for 10 min and washed once using PBS buffer. Cell pellets were lysed in 500 μL of enzymatic lysis buffer (20 mM Tris HCl pH 8, 2 mM sodium EDTA, 1.2% Triton X-100, 20 mg/mL lysozyme), incubated at 37 °C for 30 min and then ultrasonicated to release the intracellular organelles and enzymes. The disrupted cells were then centrifuged (12,000× *g* for 10 min at 4 °C) and the supernatant collected. The protein content of the supernatant was determined by the Lowry method [20]. For the D-lactate dehydrogenase activity, 50 µg of protein lysate was transferred in a 1 mL cuvette and added to 20 µL of D(−)-lactic acid 100 mM and 10 µL of NAD^+^ 200 mM, and the final volume was adjusted to 1 mL, adding phosphate buffer 100 mM pH = 7.8. The enzyme activity was evaluated spectrophotometrically with the NADH absorbance at 340 nm (ε_340nm_ = 6220 M^−1^ cm^−1^) using a spectrophotometer (Jasco V-550) equipped with a thermostatic control and stirring device.

### 2.6. Lactate Determination

Lactobacilli cultures were adjusted to an OD_600nm_ of 2.0 with sterile phosphate buffer solution (PBS) (cell concentration: 5 × 10^8^ CFU/mL) and centrifuged at 5000× *g* for 10 min at 4 °C. Supernatants were filtered through a 0.2 μm membrane filter to obtain stock cell-free supernatants.

Prior to injection, the supernatants were diluted 1:10 in mobile phase and centrifuged at 14,000× *g* for 5 min at 4 °C. The supernatant was then injected manually into the HPLC system (Agilent 1100 series). Metabolites were separated on a C18 column (Agilent ZORBAX SB-Phenyl, 5 μm, 250 Å, 4.6 mm), using a mobile phase consisting of 50 mM KH_2_PO_4_, pH 2.9, at a flow rate of 0.8 mL/min. Lactate was detected using an Agilent UV detector set to 210 nm and quantified using Agilent Chem Station software (version LTS 01.11).

### 2.7. Energetic Profile

To assess the energetic profile of HeLa cells after addition of diluted supernatants or addition of D(−)- or L(+)-LA at the final concentration of 10 mM, around 7.5 × 10^3^ cells were seeded in every well of a 96-well plate. Treatment for 1 h with supernatant or LA solutions was carried out 24 h after the seeding. Cells were then washed twice with PBS, and 180 μL of fresh medium were added, then the 96-well plate was placed immediately in the Seahorse Agilent (Agilent Technologies, Santa Clara, CA, USA), and the ATPase inhibitor oligomycin A was added to evaluate the proton leak, while the uncoupler FCCP was added to obtain the maximal respiration rate.

Evaluation of the energetic profile was performed following the standard protocol of the Agilent Seahorse XF Cell Mito Stress Test.

### 2.8. Histone Post-Translational Modification

The level of histone acetylation/lactylation was determined by immunoblot analysis, a semi-quantitative technique widely used in molecular biology and biochemistry research. HeLa cells were seeded in a dish, and after 48 h they were treated for 24 h with *L. crispatus* or *L. reuteri* supernatant diluted 1:100 in culture medium. Cells were harvested and washed with 10 mM sodium butyrate in PBS, and nuclei were isolated in according to Amellem et al. [21]. The nuclear histones were extracted as previously described [22]. Histones were detected resolving samples on a 10% gel in MES buffer at 200 V for 40 min. Western Blotting was performed in transfer buffer at 100 V for 1 h. The nitrocellulose membrane was incubated with primary antibody specific for anti-acetylated lysine (Millipore, Billerica, MA, USA) for 1 h. After washes with PBS-TWEEN 20 0.1%, the membrane was incubated as before with secondary horseradish-peroxidase-conjugated antibody (GE Healthcare, Milan, Italy). After washes with PBS-TWEEN 20 0.1%, antibody binding was detected using an Amersham ECL Plus Western Blotting Detection System (GE Healthcare, Milan, Italy). Densitometry analysis was performed with a Fluor-S Max MultiImager (Bio-Rad, Hercules, CA, USA), and relative quantification of histone acetylation signals was carried out by using densitometry and normalized on H1 signal as a control.

### 2.9. Immunoblot

The level of protein expression was determined by immunoblot analysis, a semi-quantitative technique widely used in molecular biology and biochemistry research. HeLa cells were treated with *L. crispatus* or *L. reuteri* supernatant diluted 1:100 in culture medium for 48 h. Cells were washed twice with PBS and lysed for 1 h in lysis buffer (HEPES, pH 7.4 40 mM, glycerophosphate 60 mM, p-nitrophenylphosphate 20 mM, Na_3_PO_4_ 0.5 mM, NaCl 250 mM, Triton X-100 1%, PMSF 0.5 mM and 10 mg/mL each of aprotinin, leupeptin, pepstatin and antipain (Sigma-Aldrich)) at 0 °C. Cell lysates were centrifuged at 12,000× *g* for 20 min. Supernatants were collected and protein concentration determined by using the Bio-Rad protein assay method (Bio-Rad, Hercules, CA, USA). The proteins were resolved on a 7.5% or 10% polyacrylamide gel and immunoblotted with a rabbit anti-human α5 integrin subunit (Cell Signaling Technology, Danvers, MA, USA), rabbit anti-EGFR (Biorbyt, Cowley Road, Cambridge, UK), rabbit anti-cyclin D1 (Millipore, Billerica, MA, USA), rabbit anti-p21 (Millipore, Billerica, MA, USA) or mouse anti-α-tubulin (Sigma-Aldrich) antibodies. Detection of immunoreactive bands was performed by using a rabbit or mouse HRP-conjugated secondary antibody (GE Healthcare, Milan, Italy) followed by the Amersham ECL Plus Western Blotting Detection System (GE Healthcare, Milan, Italy). Densitometry analysis of immunoreactive bands was conducted with a Fluor-S Max MultiImager (Bio-Rad). Relative quantification of bands was performed by using α-tubulin signal as control. Alternatively, HeLa cells were treated with a 10 mM solution od D(−)-LA or L(+)-LA. Protein extraction and immunoblot analysis were performed as described above.

## 3. Results

### 3.1. Lactate Determination and Lactate Dehydrogenase Activity

**Figure 1:**
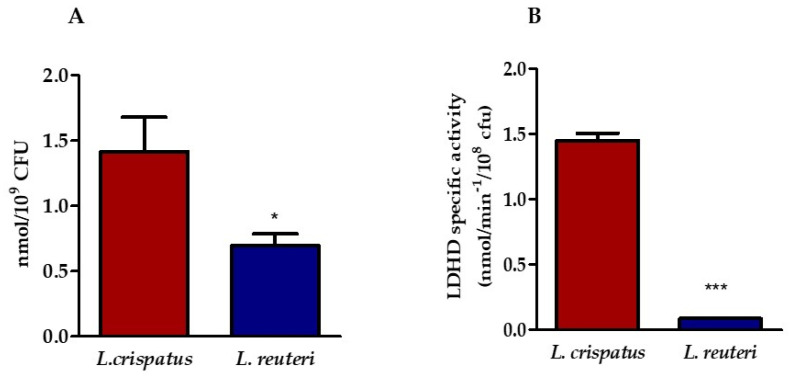
Determination of the amount of LA in lactobacilli supernatant. (**A**) HPLC analysis of LA content in the supernatants of *L. crispatus* and *L. reuteri*. Statistical significance was calculated vs. control (* *p* ≤ 0.01). (**B**) Specific activity of LDHD in bacterial lysates (*** *p* ≤ 0.0001). Results are expressed as nanomoles (nmol) of LA/mL of supernatant containing 10^9^ CFU of lactobacilli (panel **A**) and as nanomoles (nmol) of lactic acid min^−1^/10^9^ CFU (panel **B**). Bars represent mean values, and error bars represent standard deviation. All measurements were performed in duplicate on six separate occasions.

Two strains of lactobacilli belonging to two species, *L. crispatus* and *L. reuteri*, were selected for this in vitro study. The amount of LA released by the lactobacilli was determined in the supernatants of *L. crispatus* and *L. reuteri* after 24 h of culture by HPLC. Figure 1A shows that the supernatant of *L. crispatus* contains a higher amount of LA than that of *L. reuteri*. Because we were unable to determine the relative concentrations of the two LA isomers with our HPLC system, we tested the presence of the D-lactate dehydrogenase (LDHD) enzyme with kinetic analysis. To this end, we measured the activity of LDHD in the bacterial lysates in the presence of a saturating concentration of D(−)-LA, which can provide an approximate indication of the amount of the enzyme. The results shown in Figure 1B confirm the presence of LDHD in the *L. crispatus* lysate with a maximum specific activity of 1.46 nmol min^−1^/10^8^ CFU, whereas it is almost completely absent in the *L. reuteri* lysate, where the maximal specific activity was 0.08 nmol min^−1^/10^8^ CFU.

These data were confirmed by the analysis performed using the liquid chromatography–mass spectrometry technique (LC-MS). The LC-MS analysis of peptides obtained by digestion of *L. crispatus* proteins led to the identification of 114 proteins, among which was D-lactate dehydrogenase. The identified peptides are listed in the supporting information (File S1) along with the other parameters obtained by searching the SwissProt database. The same analysis performed for proteins from *L. reuteri* resulted in the identification of 92 proteins, but D-lactate dehydrogenase was not found.

### 3.2. Evaluation of Lactobacilli Cell Pellets and Supernatant’s Protective Effect against CT Infection

**Figure 2:**
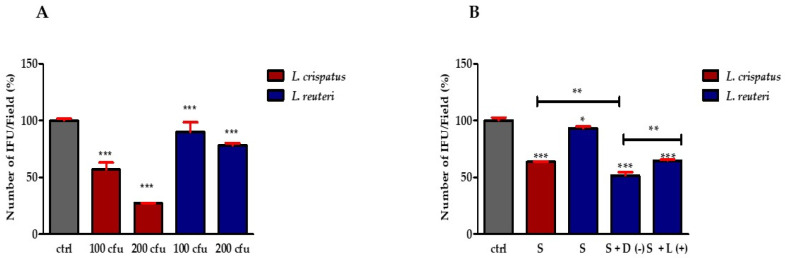
Effect of lactobacilli on CT infectivity. (**A**) Effect of lactobacilli cell pellets on CT infectivity. Quantification of HeLa cells positive to CT inclusions. The results are expressed as a percentage of CT infectivity, comparing the number of IFUs with the control (set at 100%). Bars represent mean values, and red error bars represent standard deviation. (**B**) Percentage of CT-infected HeLa cells pre-exposed to *L. crispatus* or *L. reuteri* culture supernatant (S) (diluted 1:100) and *L. reuteri* culture supernatant supplemented with 10 mM D(−)- or L(+)-LA. Results are from three independent experiments. Statistical significance is calculated vs. control (* *p* ≤ 0.01; ** *p* ≤ 0.001; *** *p* ≤ 0.0001). All measurements were performed in duplicate on six separate occasions.

To evaluate the role of lactobacilli in preventing CT infection, two different lactobacilli fractions (cell pellets and supernatant) were tested. First, HeLa cells were incubated with *L. crispatus* or *L. reuteri* at a ratio of 1:100 or 1:200 (HeLa cells: *lactobacilli*) for 1 h and then treated with 5 × 10^4^ CT EBs for 1 h (exclusion mechanism). As shown in Figure 2A, treatment with *L. crispatus* reduced the infectivity of CT by 36.4% ± 1.65 (1:100 ratio) and 72.7% ± 0.36 (1:200 ratio), respectively, whereas *L. reuteri* was less active in protecting against infection.

To distinguish the effect of lactobacilli from the effect of secreted molecules produced by bacteria during growth and to evaluate the effect of the two LA isomers, we tested the effect of bacterial supernatants and the additive effect of D(−)- or L(+)-LA on the infectivity of CT in HeLa cells. Before investigating the protective effect of *Lactobacillus* supernatants and LA isomers, we performed preliminary experiments to determine the concentrations to be used. For this purpose, we treated HeLa cells with different dilutions of supernatants from *L. crispatus* and *L. reuteri* cultures. The results shown in Figure S2A,B (File S2) indicate that the 1:100 dilution of the two bacterial supernatants is safe for HeLa cells. At the same time, we tested the effect of the two LA isomers on cell viability. The results shown in Figure S2C (File S2) indicate that both isomers of LA have very low cytotoxicity at a concentration of 10 mM for 24 h of treatment. Finally, we tested the effect of 24 h treatment with 1:100 diluted *L. reuteri* supernatant enriched with different concentrations of LA isomers (1, 5 and 10 mM) on HeLa cell viability. Figure S2D (File S2) shows that 10 mM of D(−)-LA has no significant effect on cell viability, while L(+)-LA reduces it by about 20%.

Based on these results, we treated HeLa cells with 1:100 diluted supernatants for 1 h, then the medium was removed, and the cells were treated with 5 × 10^4^ CT EBs for 1 h. The results shown in Figure 2B indicate that the protective effect of *L. crispatus* supernatants was maintained. In particular, treatment with *L. crispatus* supernatant reduced the infectivity of CT by 37.08% ± 0.35, whereas treatment with *L. reuteri* had a much lower inhibitory effect against CT (the reduction only being equal to 7.01% ± 1.54). On the other hand, the addition of 10 mM D(−)-LA to the *L. reuteri* supernatant increased its resistance against infection (infectivity reduction rose to 48.33% ± 2.89), while the addition of 10 mM L(+)-LA led to a significantly lower infectivity reduction (35.16% ± 0.81) (Figure 2B). These results suggest that D(−)-LA plays a central role in reducing CT infectivity.

**Figure 3:**
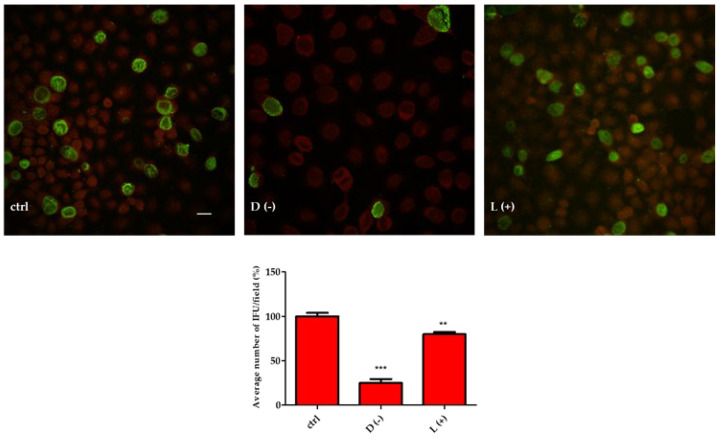
D(−)-LA prevents CT infection in HeLa cells. (**Above**). HeLa cells were incubated or not with 10 mM of D(−)- or L(+)-LA for 1 h and then with CT EBs for an additional hour. Specimens were stained for chlamydial membrane lipopolysaccharide antigen (green fluorescence). Representative micrographs are shown. Bar: 20 µm. (**Below**). Percentage of CT infected HeLa cells. Results are from three independent experiments compared to control. (** *p* ≤ 0.01; *** *p* < 0.0001).

To improve this point, we preloaded HeLa cells with 10 mM of D(−)-LA or L(+)-LA prior to incubation with 5 × 10^4^ CT EBs. The results shown in Figure 3 indicate that infection of CT was greatly reduced by D(−)-LA treatment.

### 3.3. Oxygen Consumption Rate in HeLa Cells Treated with L. crispatus or L. reuteri Supernatants

**Figure 4:**
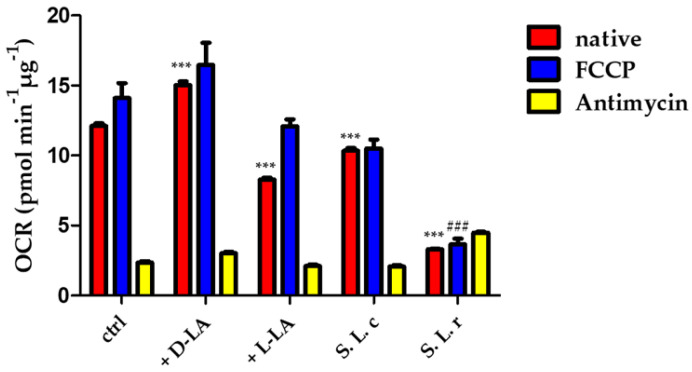
Oxygen consumption rate (OCR) was measured in HeLa cells (ctrl) and in HeLa incubated with 10 mM of D(−) or L(+)-LA or treated for 1 h with *L. crispatus* (S *L.c.*) and *L. reuteri* (S *L.r.*) supernatant following the addition of the OXPHOS uncoupler FCCP (0.5 µM) and the electron transport inhibitor antimycin A (AA) (2 µM). Statistical significance of native OCR (red bars) is calculated vs. control (ctrl) (*** *p* ≤ 0.0001); the uncoupled respiration rate (blue bars) is significantly decreased by the treatment with *L. reuteri* supernatant (^###^ *p* ≤ 0.0001). Data are mean ± SD.

The oxygen consumption rate (OCR) is an indicator of the metabolic state of the cell: cells with a more oxidative metabolism are characterized by a higher oxygen consumption rate, whereas a low oxygen consumption rate indicates a more glycolytic metabolism. To investigate the effect of D(−)- or L(+)-LA on the metabolic status of HeLa cells, we measured the OCR of cells treated with 10 mM D(−)- or L(+)-LA compared with the effect of treatment with *L. crispatus* or *L. reuteri* supernatants. The results shown in Figure 4 indicate that OCR in HeLa cells was stimulated by supplementation with D(−)-LA. On the other hand, addition of *L. crispatus* supernatant caused a slight decrease in OCR of HeLa, whereas addition of the L(+)-LA or addition of *L. reuteri* supernatant caused a strong decrease in both native and uncoupled OCR. This result suggests that HeLa cells exhibit a more oxidative metabolism in the presence of the D(−)-LA isomer, whereas the L(+)-LA isomer stimulates a more glycolytic metabolism.

### 3.4. Lactobacilli Culture Supernatants Modulate Histone Modification State and Gene Expression

**Figure 5:**
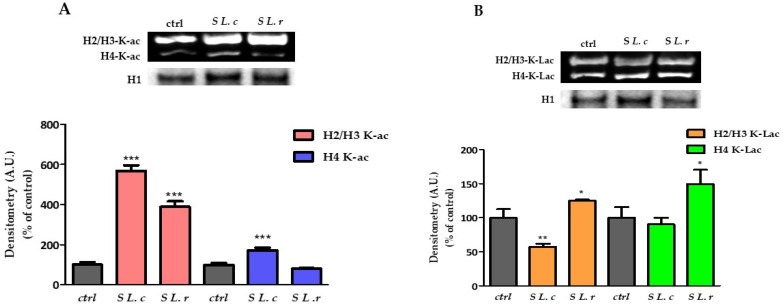
Changes in the state of histone acetylation and lactylation. (**A**) (Above). Western blot of acetylated histones. (Below). Relative quantification of H2/H3 acetylated histones in HeLa control and exposed for 24 h to culture supernatants of *L. crispatus* (S *L.c*) or *L. reuteri* (S *L.r*). (**B**) (Above). Western blot of lactylated histones. (Below). Relative quantification of H4 lactylated histones in HeLa control and treated with culture supernatants of *L. crispatus* or *L. reuteri* for 24 h. Densitometry arbitrary units (A.U.) are normalized by H1 histone. Results are given as means ± SD of three independent experiments and are compared to controls, taken as 100%. (* *p* < 0.05; ** *p* < 0.001; *** *p* < 0.0001).

Histones can be modified by cellular enzymes that add chemical marks such as methyl, acetyl and phosphate groups; these epigenetic modifications of the genome affect processes such as gene expression and DNA replication and repair [23]. Lactic acid can promote histone acetylation by inhibiting histone deacetylase (HDAC), thereby regulating gene expression [24]. In addition, it has recently been shown that LA can induce a particular type of histone modification called “lactylation” [25]. In this section, we analyzed the effect of the two isomers LA and the supernatants of *L. crispatus* and *L. reuteri* on histone modifications and gene expression in HeLa cells. HeLa cells exposed to culture supernatants of *L. crispatus* or *L. reuteri* for 24 h showed a significant increase in histone acetylation. Specifically, treatment with *L. crispatus* supernatant induced a greater increase in H2/H3 histone acetylation (~6-fold compared to a less than 4-fold increase induced by the addition of *L. reuteri* supernatant) (*p* < 0.0001) and a strong acetylation of histone H4 (+72.6%). Treatment with *L. reuteri* supernatant had no effect (Figure 5A). Interestingly, supernatant of both lactobacilli led to changes in the state of histone lactylation: supernatant of *L. crispatus* decreased lactylation of H2/H3 by 41.7% ± 12.76% and did not lead to changes in H4 histones. In contrast, the supernatant of *L. reuteri* significantly increased the lactylation of H2/H3 and H4 histones by approximately 26.1% ± 15.37 and 52.5% ± 34.59, respectively (Figure 5B).

These results suggest that *L. crispatus* and *L. reuteri* induce different epigenetic alteration patterns in HeLa cells via their metabolites, which could lead to differential regulation of gene expression. Based on this observation, we performed qtRT-PCR analysis of genes involved in cell-cycle regulation and genes such as HER.1 and ITAG5, which encode membrane proteins required for the internalization of chlamydial EBs into host cells. The qtRT-PCR results were confirmed by immunoblot analysis of the corresponding host proteins.

It is known that proteins on the cell membrane, such as integrin α5β1, are important for CT entry into the cell or development of infection [12]. In addition, downregulation of cell-cycle regulators appears to be involved in *C. trachomatis* infection [26]. The transcription levels of CCND1 (cyclin D1), CDKN1A (p21), HER-1(EGFR) and ITAG5 (α5β1) were analyzed by qtRT-PCR on cDNA of HeLa control cells treated with diluted supernatants of *L. crispatus* or *L. reuteri* for 24 h. The results presented in Figure S3 (File S3) show that the supernatant of *L. crispatus* caused a strong downregulation of CCND1, Her-1 and ITAG5 genes (0.47 ± 0.01; 0.29 ± 0.01 and 0.53 ± 0.05, respectively) (*p* ≤ 0.001 compared to control); on the other hand, CDKN1A transcription was significantly more upregulated in cells treated with *L. reuteri* supernatant by a factor of 2.55 ± 0.063 (*p* ≤ 0.001 compared to control).

**Figure 6:**
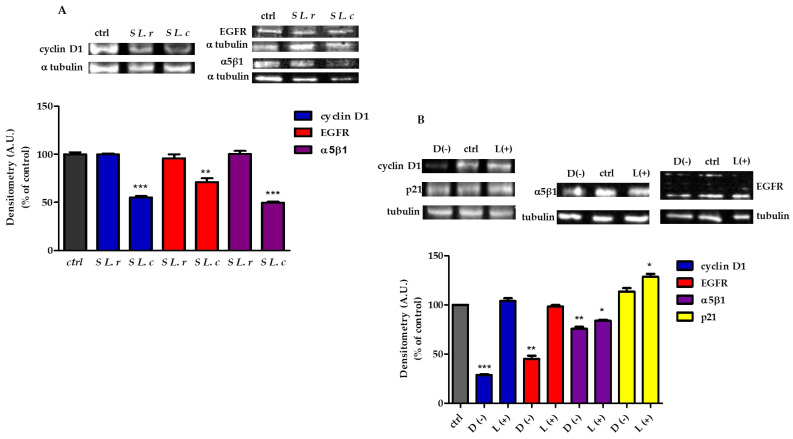
Protein expression modifications by supernatants of *L. crispatus* (S *L.c*) or *L. reuteri* (S *L.r*) modified protein expression. (**A**) (Above). (sx) Immunoblot analysis of cyclin D1 protein and (dx) Western blot of EGFR and α5β1 subunit of integrin 1. (Below). Relative quantification of proteins. Densitometry arbitrary units (A.U.) were normalized by α-tubulin. Results are given as means ± SD of three independent experiments and are compared to controls (statistical significance: ** *p* ≤ 0.001; *** *p* ≤ 0.0001), taken as 100%. (**B**) HeLa treated with 10 mM of D(−)- or L(+)-LA. (Above). (sx) Immunoblot analysis of cyclin D1 and p21 proteins and (dx) Western blot of EGFR and α5β1 subunit of integrin 1. (Below). Relative quantification of proteins. Densitometry arbitrary units (A.U.) were normalized by α-tubulin. Results are given as means ± SD of three independent experiments and are compared to controls (statistical significance: * *p* ≤ 0.01; ** *p* ≤ 0.001; *** *p* ≤ 0.0001), taken as 100%.

To validate the qtRT-PCR results, we examined the expression of the corresponding proteins by immunoblot analysis. The results shown in Figure 6 demonstrated a decrease in the expression of EGFR, cy-clin D1 and α5β1-integrin in HeLa cells treated with the supernatant of *L. crispatus* (Figure 6A) or 10 mM of D(−)-LA (Figure 6B). EGFR expression was greatly decreased by treatment with 10 mM of D(−)-LA (Figure 6B). On the other hand, the expression of p21 was increased only in cells treated with the supernatant of *L. reuteri* and was significantly increased in HeLa cells treated with 10 mM of L(+)-LA (Figure 6B).

## 4. Discussion

In this work, two different species of *lactobacilli* (*L. crispatus* and *L. reuteri*) were tested to investigate their protective effect against CT infection.

The differences that characterize these two bacterial strains include the amount and type of LA produced, which is the focus of this study. *L. crispatus* was chosen because it is known to be able to produce both isomers of LA. In contrast, *L. reuteri*, which is known to be very abundant in the gut, is a far less effective producer of D(−)-LA [15]. In particular, the isolate used in the present study produces only L(+)-LA isomer. Although *L. reuteri* is less abundant in vaginal fluids, it is more stable and easier to culture than the other lactobacilli species that colonize the vaginal environment.

LA is one of the most important antimicrobial compounds produced by vaginal lactobacilli [9], so we first investigated its content in the free lactobacilli supernatants using HPLC. The results showed that the amount of LA produced by *L. crispatus* was higher than the amount produced by *L. reuteri*.

These results are consistent with the stronger protective function of *L. crispatus* reported in the literature [10,11,12,16,17,27]. To confirm the ability of *L. crispatus* and *L. reuteri* to produce the D(−)-LA isomer, we assessed the presence of D-lactate dehydrogenase by LC-MS analysis and performed a kinetic assay to measure the maximum activity of the enzyme. We were able to demonstrate that only *L. crispatus* can produce the D(−)-LA isomer.

The mechanisms underlying the protective properties of LA are multiple: (i) the protonated form of LA can penetrate cell membranes and acidify the cytosol; (ii) LA can also weaken the bacterial cell wall or affect host cell membranes, reducing their susceptibility to infection and finally (iii) LA can induce epigenetic changes that could affect target cell gene expression [9,13,14]. These mechanisms independently or together determine host susceptibility and consequently the relationship between host and microbiota.

In terms of activity against CT infection, vaginal lactobacilli can be classified into strains with high and low protection. In the work of Edwards et al. [28] it is reported that lactobacilli producing only the L(+)-LA belong to the low activity group, suggesting that D(−)-LA producers provide higher protection against CT infection. To test this hypothesis, additional experiments were performed by adding 10 mM of D(−)-LA or L(+)-LA to *L. reuteri* supernatant, which was subsequently used to pretreat HeLa cells before CT infection. The addition of D(−)-LA resulted in enhanced protection against CT infection, comparable to the protection exerted by *L. crispatus* supernatant.

Since the acid-base properties of the two isomers are quite similar, the higher anti-chlamydial activity of D(−)-LA is likely due to the presence of specific targets. Edwards et al. suggested that D(−)-LA might modulate vaginal cell homeostasis through epigenetic modifications [28]. Therefore, we performed an in-depth analysis of epigenetic modifications of histone proteins and expression of genes involved in the regulation of cell proliferation.

HeLa cells exposed to *L. crispatus* supernatant for 24 h showed a significant increase in H2/H3 and H4 histone acetylation, whereas treatment with *L. reuteri* supernatant had only a minor effect on H2/H3 acetylation and no effect at all on H4 acetylation. It is well known that histone lysine acetylation plays an important role in epigenetic regulation of gene expression by decondensing nucleosome structure [26,29], which alters histone–DNA interactions [30], facilitating access and binding of transcription factors to genes [31,32].

Zhang et al. recently found that lactic acid can also alter the lysine residues of histones in a new epigenetic modification, lactylation, which directly stimulates gene transcription from chromatin [25]. The alteration of gene expression induced by epigenetic changes may provide further clues to explain the protective effect of D(−)-LA. For this reason, the supernatant-induced epigenetic changes were studied in relation to the state of acetylation and lactylation of histone proteins.

Lactate is an end product of anaerobic glycolysis, whose intracellular concentration increases during the Warburg effect and is responsible for several changes involved in cell survival under unfavorable environmental conditions. It has been suggested that the excess of lactate could be used to introduce epigenetic changes. Zhang et al. [25] showed that lysine histone lactylation stimulates gene transcription, suggesting a novel role of lactate in various pathological conditions such as infections or cancer. Recently, Moreno-Yruela et al. [33] reported that lysine histone lactylation is mediated by histone acetyltransferases such as p300, whereas HDAC 1-3 are responsible for lysine histone delactylation. Although both isomers are present in cells, the L(+)-LA isomer is preferred as a metabolic substrate and signaling molecule. The preferential use of the L(+)-LA isomer is mainly due to two factors: the concentration of the L(+)-LA is more than a thousand times higher than that of the D(−)-LA form [34], and L(+)-lactate is also recognized as a precursor for lysine lactylation [27]. The D(−) form of lactic acid may increase under certain conditions, such as the activation of the glyoxalase pathway observed in some tumor cells [35,36], or may be overproduced by certain microorganisms. Our hypothesis is that the presence of high levels of D(−)-LA influences epigenetic histone modifications that favor a cellular phenotype more resistant to CT infection.

Interestingly, we found that treatment of HeLa cells with the supernatants of both lactobacilli induced changes in the state of histone lactylation, suggesting that they may activate different epigenetic pathways. Indeed, treatment with the supernatant of *L. crispatus* resulted in downregulation of genes involved in cell-cycle regulation (CCND1, CDKN1A and HER-1) and significant downregulation of the ITGA5 gene encoding the α5β1 integrin. Immunoblot analysis of protein expressions of cyclin D1, p21, EGFR and α5β1 confirmed the results of RT-qPCR. Interestingly, treatment with 10 mM D(−)-LA induced decreased protein expression as observed in HeLa cells treated with *L. crispatus* supernatant. Edwards et al. [28] mentioned that the vaginal microbiota can counteract susceptibility to CT infection by controlling the cell cycle. Their results suggest that lactobacilli producing D(−)-LA downregulate the proliferation of vaginal epithelial cells, protecting them from CT infection. Other authors showed that culture supernatants of *L. crispatus* and *L. jensenii* modulated the expression of several genes related to cell proliferation, including a decrease in CCND1 and HER-1. In contrast, *L. iners*, which is unable to produce D(−)-LA, failed to downregulate these genes and protect against infection with CT [15,28]. Moreover, previous studies have shown that EGFR is required for the internalization of chlamydial EBs into host cells [37]. Parolin et al. demonstrated that infection of CT was greatly reduced when a specific blocking antibody masked the α5-integrin subunit or the corresponding gene expression was suppressed, supporting the hypothesis that the α5-subunit is critical for infection of CT in HeLa cells [12].

CT is an obligate intracellular bacterium with a very small (~1.04 Mb) genome [38] that lacks several genes for metabolic enzymes. For this reason, CT is strongly influenced by host metabolic conditions. Since CT lacks hexokinase, EBs and RBs require glucose-6 phosphate. Moreover, during the transition from EB to RB, CT is unable to meet the increased demand for ATP, making RB completely dependent on host ATP as an energy source [39,40]. Rother et al. [41] studied the changes in host metabolism following infection with CT and found that many glycolytic metabolites and TCA intermediates were upregulated in a manner similar to Warburg metabolism.

We examined the metabolic profile of HeLa cells before and after 24 h treatment with the lactobacilli supernatants using the Seahorse Flux Analyzer. Our results showed that HeLa cells treated with *L. reuteri* supernatant or 10 mM L(+)-LA for 24 h were characterized by a strong decrease in OCR profile, indicating a shift from oxidative to glycolytic metabolism that could promote CT infection. In contrast, both treatment with *L. crispatus* supernatant and with 10 mM D(−)-LA did not alter the metabolic profile of HeLa.

The lack of knowledge of the relative amounts of D(−)-LA and L(+)-LA present in the *L. crispatus* supernatant is a limitation of this work and may account for the differences in results observed when HeLa cells were treated with a 10 mM solution of the pure LA isomers. At the same time, another limitation concerns the use of two clinical isolates of *Lactobacillus*: we are fully aware that there are several strain-specific characteristics that can prevent a complete replication of the above listed results. However, treatment of the cells with the pure LA isomers replicated the effect of the supernatants of the two lactobacilli used.

In conclusion, our results demonstrate the stronger effect of D(−)-LA compared to L(+)-LA in protecting the host cell and provide new perspectives for the selection of probiotic lactobacilli strains for the prevention of chlamydia infections.

## Data Availability

All relevant research data are presented in the text or in the Supplementary Materials.

## Supplementary Materials

The following supporting information can be downloaded at: https://www.mdpi.com/article/10.3390/pathogens12070883/s1, File S1: LC-ESI-MS/MS analysis of protein from *L. crispatus* and *L. reuteri*; File S2: The effect on cell viability of pre-exposing host cells to lactic acid or *Lactobacillus* culture supernatants; File S3: The effect on gene expression of pre-exposing the host cells to *Lactobacillus* culture supernatants.
