# Supplementary material for: Role of D(−)-Lactic Acid in Prevention of Chlamydia trachomatis Infection in an In Vitro Model of HeLa Cells
Source: Pathogens. 2023 Jun 28;12(7):883. doi: 10.3390/pathogens12070883 (PMC10383594; doi:10.3390/pathogens12070883)
Supplement: Supplementary file 1 [file pathogens-12-00883-s001.zip › pathogens-2329861-supplementary.pdf]

SUPPORTING INFORMATION

Role of D(-)-lactic acid in prevention of *Chlamydia trachomatis* infection in an *in vitro* model of HeLa cells.

Chiara Zalambani, Nicola Rizzardi, Giacomo Marziali, Claudio Foschi, Sara Morselli, Marielle Ezekielle Djusse, Marina Naldi, Romana Fato, Natalia Calonghi, and Antonella Marangoni

File S1: LC-ESI-MS/MS analysis of protein from L. crispatus and L. reuteri

Sample preparation. Fifty µL of ammonium bicarbonate buffer (50 mM, pH 8.0) were added to the protein extracts from *L. crispatus* and *L. reuteri* culture, and briefly vortexed. Five µL of dithiothreitol 0.1 M in ammonium bicarbonate were added and samples were incubated at 56°C for 30 min under agitation (400 rpm). Afterwards, 10 µL of a 10 mg/mL solution of iodoacetamide in ammonium bicarbonate 50 mM, pH 8, were added and samples were incubated for 40 min in the dark at room temperature. Finally, an aliquot (1 µL) of a 1 mg/mL trypsin solution in HCl 2 mM was added and the samples were incubated at37 °C, under gentle agitation (400 rpm) overnight (18 h). Digestion was stopped by adding 2 µL of a 10% aqueous solution of formic acid. Ten µL of tryptic digest were analyzed by LC-ESI-MS/MS.

LC-ESI-MS/MS analysis

Chromatographic separation of tryptic digests was carried out by using an ACQUITY Quaternary Solvent Manager (Waters, Manchester, UK) equipped with an autosampler. Analyses were performed on a C18 (Acquity, UPLC, BEH;100×2.1 mm i.d., 1.7 µm) column. Mobile phases A (water /FA, 100/0.1) and B (AcCN /FA, 100/0.1) were used to develop a gradient. The solvent gradient was set as follows: 5–50% B, 25 min; 50-80% B, 1 min. The column was equilibrated with initial conditions for 3 min before the next injection. Mass spectrometry analyses were performed on a Xevo G2-XS QTof (Waters, Manchester, UK) with Z-spray ion source. The ESI-QToF source temperature was set at 120°C, the desolvation temperature at 300°C, the capillary voltage at 3.0 kV, and the cone voltage at35 V. Peptide ions within a m/z 400–2000 survey scan mass range were analyzed for subsequent fragmentation. 2+, 3+and 4+ charged ions were selected for MS/MS analyses. From a single survey scan 20 ions were selected for subsequent fragmentation. Scan returned to mass survey mode after 15 s. Scan time was 0.2 s for the parent ion and 0.1 s for the MS/MS ions. Collision energy was selected using charge state recognition.

Data processing.

The parent and fragment ion spectra obtained from LC-ESI-MS/MS analyses were processed using Mascot Distiller 2.5.1.0 (Matrix Science, London, UK), a software program that reduces MS raw data to high-quality peak lists for database searching. LC-MS/MS data were analyzed by searching the human SWISSPROT database (2021_03, 565254 sequences; 203850821 residues; http://www.uniprot.org) selecting bacteria (eubacteria) as taxonomy and allowing only three missed cleavages. The precursor and fragment ion tolerance were 0.3 and 0.3 Da, respectively. Cysteine carbamidomethylation was selected as fixed modification while methionine oxidation was selected as variable modification.

Results.

LC-MS analysis of peptides obtained by the digestion of proteins from *L. crispatus* led to the identification of 114 proteins, includingthe D-lactate dehydrogenase. The identified peptides along with the other parameters achieved by SwissProt database search are reported in table 1.

Table S1. Results from SwissProt database searching. Identified peptides along with their observed and expected molecular weight, mass error (delta), score, rank, univocity and sequences are reported.

Peptide	Observed	Mr(expt)	Mr(calc)	Delta	Score	Rank	U	Peptide
11-21	717.9360	1433.9360	1433.6827	0.1748	84	1	U	R.KDEEPFLNEWK.E
26-42	969.1105	1936.2065	1936.9677	0.2388	15	1	U	K.DIDVDYTDKLLTPETAK.L
78-88	610.3540	1218.6935	1218.5551	0.1385	11	1	U	R.NVGVDNIDMDK.A
146-163	950.1102	1898.2058	1897.9833	0.2225	21	1	U	R.DQVVGVVGTGHIGQVFMR.I
146-163	633.7457	1898.2154	1897.9833	0.2321	55	1	U	R.DQVVGVVGTGHIGQVFMR.I
164-171	852.5232	851.5160	851.4211	0.0948	14	3	U	R.IMEGFGAK.V
164-171	1426.7667	851.5189	851.4211	0.0978	26	1	U	R.IMEGFGAK.V
227-236	559.8474	1117.6802	1117.5550	0.1252	53	1	U	K.DGVVIVNCSR.G
227236	1118.6879	1117.6806	1117.5550	0.1256	49	1	U	K.DGVVIVNCSR.G
313-319	410.7602	819.5058	819.4239	0.0819	14	3	U	K.AFNNNLK.L

The same analysis performed on proteins from *L. reuteri* led to the identification of 92 proteins, but the D-lactate dehydrogenase was not found.

File S2: The effect on cell viability of preexposing host cells to lactic acid or Lactobacillus culture supernatants

HeLa cell viability assay.

Hela cell viability was investigated by MTT assay. Briefly, the cells were exposed to media containing D(-)-LA or L(+)-LA (10, 20, 40 mM) or diluted culture supernatants (1:1, 1:10, 1:100,1:1000) or supernatant of *L. reuteri* diluted 1:100 enriched with lactic acid isomers (1, 5, 10 mM) for up to 48 hours. After treatment, the culture medium was replaced with 0.1 mL of 3-(4,5-dimethylthiazolyl-2)-2,5-diphenyltetrazolium bromide (MTT, Sigma-Aldrich, Milan, Italy) dissolved in PBS at the concentration of 0.2 mg/mL, and samples were incubated for 2 h at 37°C. The formazan salt crystals formed were dissolved with 0.2 mL of isopropyl alcohol for 20 min. The absorbance at 570 nm was measured using a multiwell plate reader (Tecan, Männedorf, Switzerland), and data were analyzed by Prism GraphPad software. Cell viability was determined as follows: viability (percentage of control)=[(absorbance sample−absorbance blank)/[absorbance control−absorbance blank)]×100.

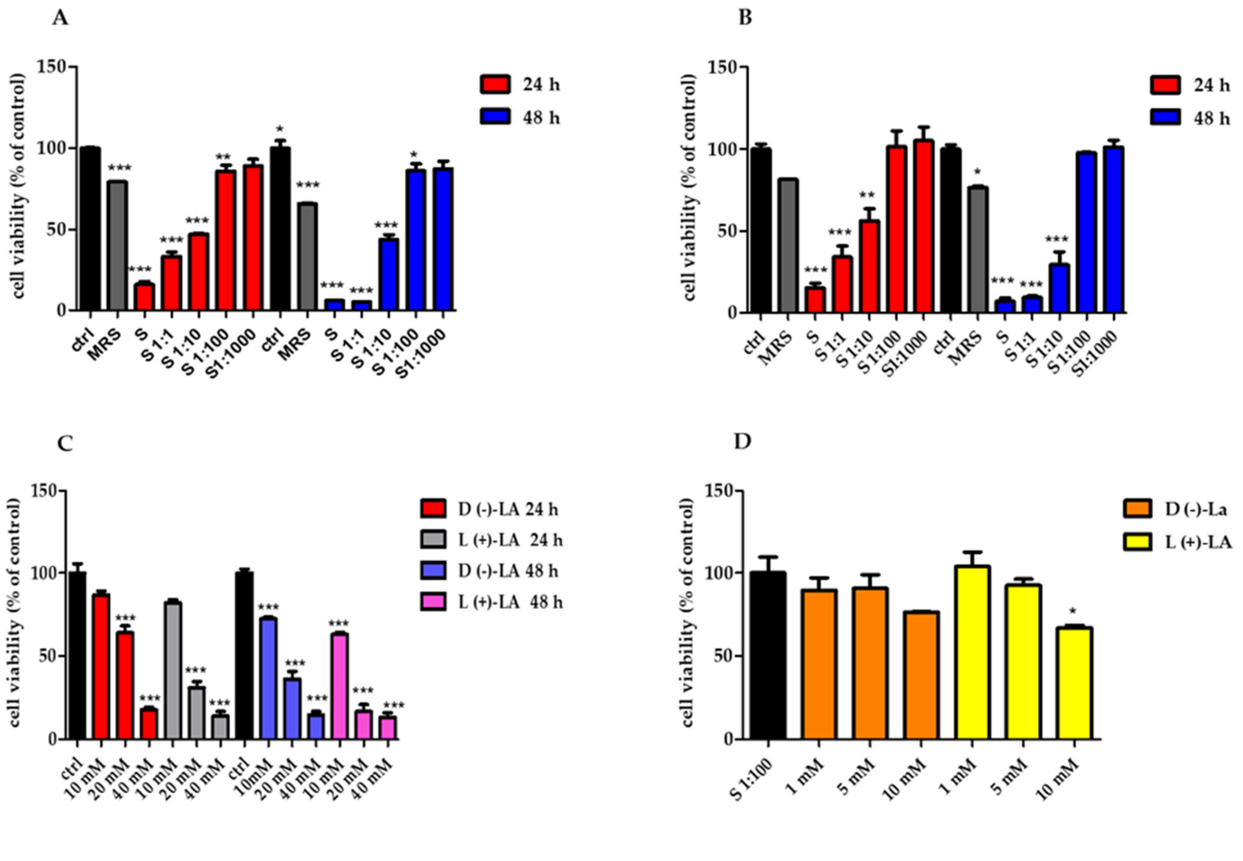

Figure S2. Effect of lactic acid and *Lactobacillus* culture supernatants on HeLa cells. Percentage of HeLa cells exposed to *L. crispatus* (A) or *L. reuteri* (B) culture supernatants diluted 1:1, 1:10, 1:100 and 1:1000 for 24 h and 48 h. (C) Percentage of HeLa cells exposed to D(-)- or L(+)-LA at concentrations of 10,20 and 40 mM for 24 and 48 hours. (D) Percentage of HeLa cells treated with L. reuteri

supernatant diluted 1:100 and enriched with D(-)-LA or L(+)-LA at concentrations of 1, 5 and 10 mM for 24 and 48 hours. The results come from three independent experiments. Statistical significance is shown as follows: *p-value <0.05; **p-value <0.01; *** p < 0.001.

File S3: The effect on gene expression of pre-exposing host cells to Lactobacillus culture supernatants

Quantitative Real-Time PCR

HeLa cells treated with *L. crispatus* or *L. reuteri* supernatants diluted 1:100 in culture medium for 24 hours. Total RNA was isolated by RNeasy Mini kit (Qiagen, Hilden, Germany) according to the manufacturer's protocol. 1 μg of RNA was reverse-transcribed with RevertAid First Strand cDNA Synthesis Kit (Fermentas, Ontario, Canada) by using oligo(dT) primers. The cDNA was analyzed by quantitative Real Time PCR (qRT-PCR), by employing the LightCycler FastStart DNA Master SYBR Green I Kit and the LightCycler 2.0 instrument (Roche Diagnostics, Manheim, Germany). Gene expression was quantified by ΔΔCT method, by using β actin as the housekeeping gene. Amplicon specificity was verified by first-derivative melting curve analysis and agarose gel electrophoresis.

Table S3: List of primers for quantitative RT-PCR.

PRIMERS	SEQUENCE
β actin FW<br>*β actin REV*	CCAACCGCGAGAAGATGA<br>CCAGAGGCGTACAGGGATAG
p21 WAF1 FW<br>*p21 WAF1 REV*	CCTAAGAGTGCTGGGCATTTT<br>TGAATTTCATAACCGCCTGTG
CCND1 FW<br>*CCND1 REV*	GCCAACTGGTGTTTGAAAGTA<br>TCCGGTGTGAAACATCTAAGA
HER-1 FW<br>*HER-1 REV*	AGCGTGAGGATTCCCGTAGCTCT<br>GAGGCAAAATGTCTACTCTCCAGGC
ITAG5 FW<br>*ITAG5 REV*	GGCAGAAGGCAGCAATGGTG<br>AGGCATCTGAGGTGGCTGGA

Results

Transcription levels of CCND1 (Cyclin D1), CDKN1A (p21), HER-1(EGFR) and ITAG5 ($\alpha 5\beta 1$) were analyzed by Real Time PCR performed on cDNA of HeLa control and treated with *L. crispatus* or *L. reuteri* diluted supernatants for 24 hours. Genes transcription was normalized to the reference gene β-actin and the relative variations (fold change) were reported. CCND1, HER-1 and ITAG5 genes expression were significantly downregulated in HeLa cells treated with *L. crispatus* supernatant (0.47 ± 0.01; 0.29 ± 0.01 and 0.53 ± 0.05 respectively) ($p \leq 0.001$ vs control); on the other hand, CDKN1A transcription was more significantly upregulated in cells treated with *L. reuteri* supernatant by a factor of 2.55 ± 0.063 ($p \leq 0.001$ vs control).

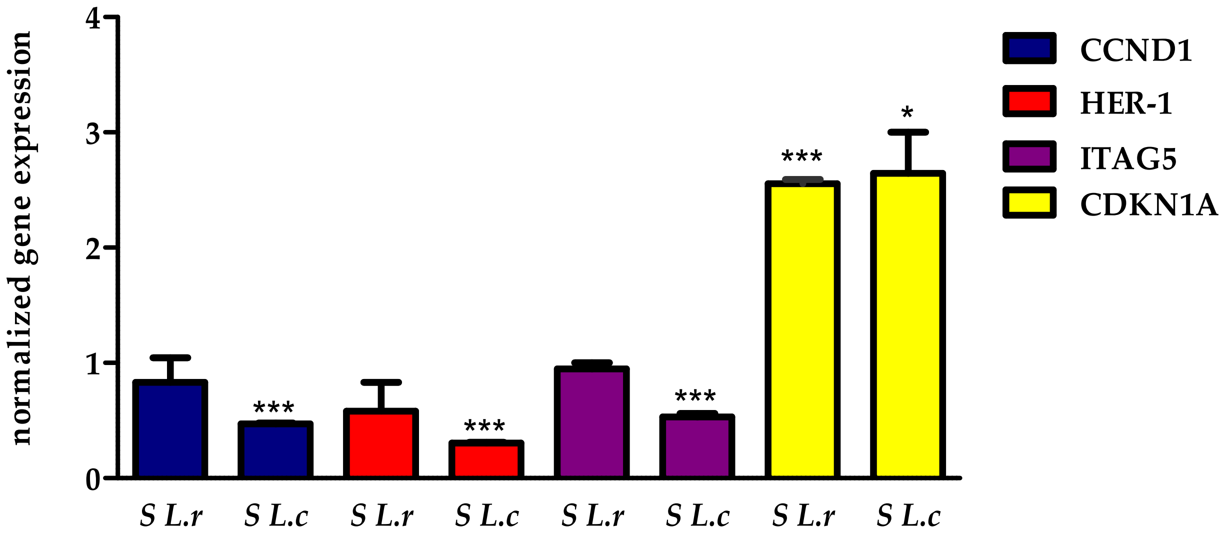

Figure S3. Supernatants of *L. crispatus* (S *L.c*) or *L. reuteri* (S *L.r*) modified gene expression. Gene expression for CCND1, HER-1, ITAG5, and CDKN1A analyzed by RT-PCR in HeLa control and treated with lactobacilli diluted supernatants for 24 hours.
